# Allometric scaling relationship between above- and below-ground biomass within and across five woody seedlings

**DOI:** 10.1002/ece3.1184

**Published:** 2014-09-27

**Authors:** Dongliang Cheng, Yuzhu Ma, Quanling Zhong, Weifeng Xu

**Affiliations:** 1 College of Geographical Science, Fujian Normal UniversityFuzhou, Fujian Province, 350007, China; 2 State Key Laboratory of Agrobiotechnology, The Chinese University of Hong KongShatin, Hongkong, 999077, China; 3 State Key Laboratory of Soil and Sustainable Agriculture, Institute of Soil Science, Chinese Academy of SciencesNanjing, 210008, China

**Keywords:** Allometry, biomass partitioning patterns, intraspecific scaling and interspecific scaling, isometric scaling, leaf, stem and root biomass allocation

## Abstract

Allometric biomass allocation theory predicts that leaf biomass (*M*_*L*_) scaled isometrically with stem (*M*_*S*_) and root (*M*_*R*_) biomass, and thus above-ground biomass (leaf and stem) (*M*_*A*_) and root (*M*_*R*_) scaled nearly isometrically with below-ground biomass (root) for tree seedlings across a wide diversity of taxa. Furthermore, prior studies also imply that scaling constant should vary with species. However, litter is known about whether such invariant isometric scaling exponents hold for intraspecific biomass allocation, and how variation in scaling constants influences the interspecific scaling relationship between above- and below-ground biomass. Biomass data of seedlings from five evergreen species were examined to test scaling relationships among biomass components across and within species. Model Type II regression was used to compare the numerical values of scaling exponents and constants among leaf, stem, root, and above- to below-ground biomass. The results indicated that *M*_*L*_ and *M*_*S*_ scaled in an isometric or a nearly isometric manner with *M*_*R*_, as well as *M*_*A*_ to *M*_*R*_ for five woody species. Significant variation was observed in the *Y-*intercepts of the biomass scaling curves, resulting in the divergence for intraspecific scaling and interspecific scaling relationships for *M*_*L*_ versus *M*_*S*_ and *M*_*L*_ versus *M*_*R*_, but not for *M*_*S*_ versus *M*_*R*_ and *M*_*A*_ versus *M*_*R*_. We conclude, therefore, that a nearly isometric scaling relationship of *M*_*A*_ versus *M*_*R*_ holds true within each of the studied woody species and across them irrespective the negative scaling relationship between leaf and stem.

## Introduction

Biomass allocation between different organs and above- versus below-ground parts is important in the performance of individual plants in terms of coping with abiotic and biotic stresses (West-Eberhard 2003; Weiner 2004; Poorter et al. 2012a), and as well as of serving community functions, such as carbon flux (Hui and Jackson 2006; Xue et al. 2013). The allometric approach for biomass allocation describes the biomass of different parts as alloemtric relationships (Enquist and Niklas 2002; Niklas 2004, 2005; Savage et al. 2008), with the mathematical formula:


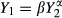


where *Y*_1_ and *Y*_2_ are biomass for different organs, *β* is a normalization (allometric) constant, and *α* is the scaling exponent. Prior work has shown that above-ground mass (leaf biomass + stem biomass, denoted by *M*_*A*_) scales, on average, nearly isometrically with respect to below-ground mass (root biomass, denoted by *M*_*R*_) across a broad spectrum of ecologically diverse vascular plants at the individual level (Enquist and Niklas 2002; Sack et al. 2002; Niklas 2004, 2005; Cheng et al. 2009; Xue et al. 2013), as well as in the community level (i.e. *α* ≈ 1.0) (Cheng and Niklas 2007; Yang et al. 2010; Yang and Luo 2011). Such isometry is predicted from a strictly analytical approach to addressing how plants annually partition their total biomass into leaf, stem and root biomass based on the assumptions of the metabolic theory of ecology (West et al. 1999).

Specifically, prior work has shown that for seedlings, which lack substantial quantities of secondary tissues, leaf, stem and root biomass should scale isometrically with respect to each other, as:


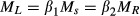
(1)

where *β* denotes an allometric constant numerically distinguished from others by its subscript. Because above-ground biomass is the sum of leaf and stem biomass: *M*_*A*_ = *M*_*L*_ + *M*_*S*_, it followings that:


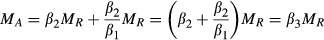
(2)

Therefore, an isometric relationship could be derived based on the isometric relationships among leaf, stem and root biomass for tree seedlings. Similarly, for larger trees, because annual accumulations of root wood exceed annual increases in leaf mass, above-ground biomass scales nearly isometrically with below-ground biomass (Enquist and Niklas 2002). Nevertheless, the isometric biomass allocation pattern in seedlings for a given species remains controversial at least for two reasons. First, despite a number of theoretical and empirical justifications for constant scaling exponents at individual and community levels across a broad range of plant taxa growing in diverse environments, the invariance of the scaling exponents has been hotly debated (e.g. Dodds et al. 2001; Kozlowski and Konarzewski 2004; Reich et al. 2006; Price et al. 2007; Koontz et al. 2009). And, second, there is no guarantee that interspecific biomass allocation patterns hold true for intraspecific biomass allocations. Specifically, ecologists have long demonstrated that ratio for above- to below-ground biomass (i.e., shoot/root ratio, SRR) varies across species and manifest adaptive responses to changes in environmental gradient (Niinemets 1998; Poorter 2001; Binkley et al. 2004; McCarthy and Enquist 2007; Cheng et al. 2009; Wang and Taub 2010; Poorter et al. 2012a). Therefore, the allometric constant, which is equal to SRR (i.e., β_3_ ≈ SRR) when above-ground biomass scales isometrically with below-ground biomass, should be expected to vary across species. Indeed, Cheng and Niklas (2007) indicated that although *M*_*A*_ scaled nearly isometrically with *M*_*R*_, scaling constants differed between forest types. In this scenario, variation in scaling constants (β) among different species might result in different scaling exponents across species. For examples, Reich et al. (2006) reported that respiration rates scales nearly isometrically with biomass in individual studies, but scales as 0.81–0.84 power of body size across all data pooled because of the variation of scaling constants among individual studies. Therefore, whether the interspecific biomass allocation patterns hold true for intraspecific biomass allocations remains to be see.

We studied scaling relationships for biomass allocation patterns among five evergreen tree seedlings to test: (1) whether the isometric scaling relationships exist among different organs (leaf, stem, and root), (2) if not, whether such allometric relationship leads to a deviation for the isometric scaling relationship between above- and below-ground biomass, and (3) how the different scaling constants influence the scaling relationship across the entire data set.

## Materials and Methods

### Study sites

The seedlings were harvested between December 2012 and April 2013 at Forestry Science and Technology Promotion Center in Shunchang County, Fujian Province, China (26°46′N, 117°52′E). Here, the climate is subtropical monsoon climate; the mean annual temperature is 18.5°C, with an average temperature of 26.85°C in the warmest month (July) and of 9.1°C in the coldest month (January); the average annual precipitation is 1756 mm and the prevalent soil type is red soil. Seedlings were sampled based on the availability in the greenhouse of Forestry Science and Technology Promotion Center, containing two gymnosperm species (i.e., *Pinus massoniana* Lamb. and *Cunninghamia lanceolata* (Lamb.) Hook.) and three angiosperm species (i.e., *Machilus pauhoi* Kanehira, *Phoebe bournei* (Hemsl.) Yang and *Schima superba* Gardn. et Champ.). The five species were the typical forest planting species in Fujian province. Specifically, mature seeds of *P. massoniana*, *C. lanceolata,* and *S. superb* were provided by forestry department of Fujian province, and seeds for *M. pauhoi,* and *Phoebe bournei* species were collected from natural populations. Before sowing, seeds were disinfected with KMnO_4_ solution for 30 min, and subsequently dipped in water at 20°C for 24 h. The dipped seeds were sown in wet sand and placed in a growth chamber until they germinated, after which they were planted individually in circle plastic containers filled with decomposed sawdust in March of 2012, expect for *S. superb*, which was planted in March of 2011. The seedlings were cultivated under sunshade net, which reduced incoming photosynthetically active radiation (PAR) by about 20% compared with that observed outside the shelter under sunny conditions. The shelter had no sidewalls, such that air temperature, wind speed, and relative humidity were similar to ambient conditions.

### Biomass measurements

The range of sizes for each species was selected to represent the whole distribution observed in greenhouse. Therefore, a total of 258 individuals, ranging in size between 0.11 and 51.39 g, and including at least 19 individuals per species, were examined. All seedlings were cut at the base of the stem, to separate above-ground parts and below-ground parts (roots), followed by separation of the above-ground parts into leaf and stem. After the soils on roots were washed out, all leaf, stem, and root parts were dried at 65°C for 72 h to determine its biomass.

### Statistical protocols

Data of leaf, stem, root, and above-ground biomass (denoted as *M*_*L*_, *M*_*S*_, *M*_*R*_, and *M*_*A*_, respectively) were log_10_-transformed. Model Type II regression was used to determine the slope (scaling exponent) and *y*-intercept (allometric constant) of log–log linear relationships (i.e., α and log β, respectively). The software package “Standardized Major Axis Tests and Routines” (Warton and Weber 2002; Falster et al. 2003) was also used to determine whether the numerical values of α for log *M*_*o*_ versus log *M*_*a*_ differed between five species, where log *M*_*o*_ and log *M*_*a*_ are the mass variables of interest (plotted on the ordinate and abscissa axis, respectively). This software package, denoted by (S) MATR, was used to provide the Model Type II equivalent of OLS standard analyses of covariance (ANCOVA). The significance level for testing slope heterogeneity was *P* < 0.05 (i.e., common slope was rejected if *P* < 0.05). If the compared regressions have common slopes but have different y-intercepts, then the difference in y-intercepts might lead to the significant difference between the common slope across species and the slope obtained from the all data.

## Results

Significant allometric relationships were detected among biomass components across and within five woody species (i.e., *r*^2^ > 0.73). For each allometry, different species typically had the nearly consistent slope with different scaling constants, except for the relationship between *M*_*L*_ versus *M*_*S*_.

### The scaling of *M*_*L*_ versus *M*_*S*_

**Figure 1:**
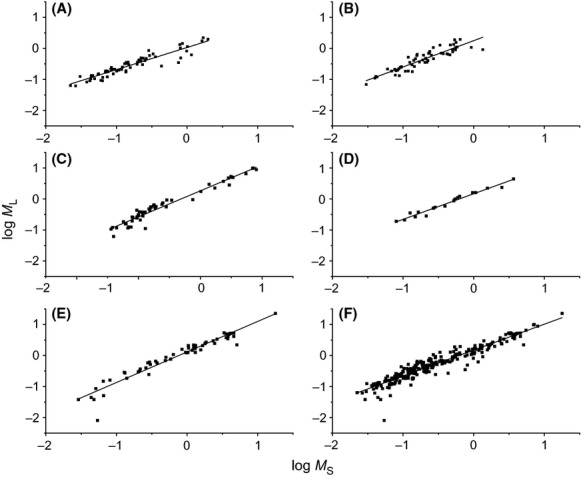
Log–log bivariate plots of leaf versus stem biomass (*M*_*L*_ vs. *M*_*S*_) within and across five evergreen tree species. (A) *Pinus massoniana*; (B) *Cunninghamia lanceolata*; (C) *Machilus pauhoi*; (D) *Phoebe bournei*; (E) *Schima superb*; (F) across five species.

The scaling exponents for leaf with respect to stem biomass differed significantly (*P* = 0.001) among five species (Table1; Fig.1). Numerically, the lowest scaling exponent was observed for *P. massoniana*; the highest was obtained for *S. superba* (i.e., α_RMA_ = 0.76 and 1.02, respectively). Based on 95% CIs overlaps and ANCOVA analyses, the *M*_*L*_ versus *M*_*S*_ scaling exponents for *C. lanceolata* and *S. superba* were statistically indistinguishable from isometry (*P* = 0.215 and 0.525, respectively), whereas the scaling exponents for the other three species were significantly <1.0 (*P* < 0.001).

**Table 1:** (S) MATR reduced major axis regression slopes and y-intercepts (α_RMA_ and log β_RMA_, respectively) for log_10_-tranformed data of leaf, stem and root (*M*_*L*_, *M*_*S*_ and *M*_*R*_, respectively), and above- and below-ground biomass (*M*_A_ and *M*_R_, respectively) within and across five species. Scaling exponents in bold type have 95% CIs that numerically include the predicted value of 1.0.

	α_RMA_ (95% CI)	log β_RMA_	*r*^*2*^
*Pinus massoniana* (*n* = 68)			
log *M*_*L*_ vs. log *M*_*S*_	0.76 (0.70; 0.83)	0.068	0.879
log *M*_*L*_ vs. log *M*_*R*_	**0.87 (0.75; 1.00)**	0.29	0.665
log *M*_*S*_ vs. log *M*_*R*_	1.14 (1.01; 1.29)	0.30	0.762
log *M*_*A*_ vs. log *M*_*R*_	**0.96 (0.85; 1.09)**	0.58	0.742
*Cunninghamia lanceolata* (*n* = 58)			
log *M*_*L*_ vs. log *M*_*S*_	**0.93 (0.83; 1.04)**	0.31	0.826
log *M*_*L*_ vs. log *M*_*R*_	**0.90 (0.79; 1.02)**	0.18	0.782
log *M*_*S*_ vs. log *M*_*R*_	**0.96 (0.84; 1.10)**	−0.14	0.739
log *M*_*A*_ vs. log *M*_*R*_	**0.91 (0.80; 1.02)**	0.35	0.798
*Machilus pauhoi* (*n* = 53)			
log *M*_*L*_ vs. log *M*_*S*_	0.86 (0.81; 0.91)	0.27	0.952
log *M*_*L*_ vs. log *M*_*R*_	**0.96 (0.88; 1.05)**	0.29	0.898
log *M*_*S*_ vs. log *M*_*R*_	1.12 (1.04; 1.20)	0.016	0.934
log *M*_*A*_ vs. log *M*_*R*_	**1.01 (0.93; 1.09)**	0.48	0.921
*Phoebe bournei* (*n* = 19)			
log *M*_*L*_ vs. log *M*_*S*_	0.84 (0.77; 0.92)	0.17	0.968
log *M*_*L*_ vs. log *M*_*R*_	**0.87 (0.73; 1.03)**	0.36	0.881
log *M*_*S*_ vs. log *M*_*R*_	**1.03 (0.89; 1.20)**	0.22	0.912
log *M*_*A*_ vs. log *M*_*R*_	**0.93 (0.79; 1.09)**	0.60	0.897
*Schima superba* (*n* = 60)			
log *M*_*L*_ vs. log *M*_*S*_	**1.02 (0.95; 1.10)**	0.12	0.926
log *M*_*L*_ vs. log *M*_*R*_	**1.00 (0.90; 1.10)**	0.13	0.862
log *M*_*S*_ vs. log *M*_*R*_	**0.97 (0.87; 1.08)**	0.0080	0.823
log *M*_*A*_ vs. log *M*_*R*_	**0.96 (0.87; 1.06)**	0.38	0.855
All data (*n* = 258)			
log *M*_*L*_ vs. log *M*_*S*_	0.88 (0.85; 0.91)	0.20	0.902
log *M*_*L*_ vs. log *M*_*R*_	0.89 (0.85; 0.93)	0.23	0.833
log *M*_*S*_ vs. log *M*_*R*_	**1.01 (0.96; 1.06)**	0.030	0.834
log *M*_*A*_ vs. log *M*_*R*_	0.92 (0.88; 0.96)	0.44	0.854

The scaling constants varied significantly among five species, ranging from 0.068 for *P. massoniana* to 0.31 for *C. lanceolata*. Therefore, *M*_*L*_ scaled as 0.88-power with respect to *M*_*S*_ across the entire data, which was significantly <1.0 expected for seedlings (*P* < 0.001 for five species).

### The scaling of *M*_*L*_ (*M*_*S*_) versus *M*_*R*_

**Figure 2:**
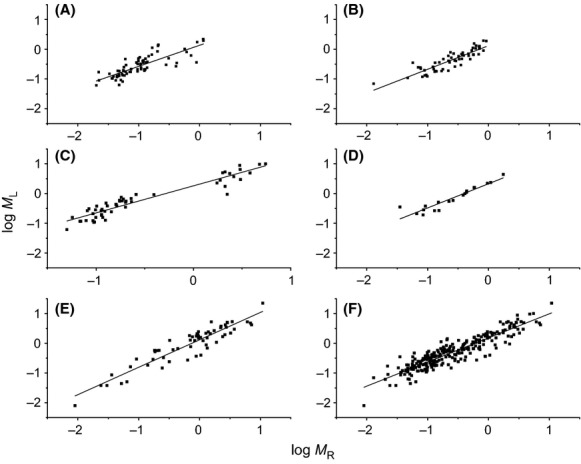
Log–log bivariate plots of leaf versus root biomass (*M*_*L*_ vs. *M*_*R*_) within and across five evergreen tree species. (A) *Pinus massoniana*; (B) *Cunninghamia lanceolata*; (C) *Machilus pauhoi*; (D) *Phoebe bournei*; (E) *Schima superb*; (F) across five species.

The isometric scaling relationship for *M*_*L*_ and *M*_*R*_ was versified for the sampled five species. Specifically, the ANCOVA results indicated that the five species had the common slope (i.e., *M*_*L*_ ∝ *M*_*R*_^0.94^, 95% CI = 0.89–0.99, *P* = 0.383) and that the scaling exponent for each species was indistinguishable from 1.0 (*P* > 0.05 for five species) (Table1; Fig.2). However, the scaling constants varied from 0.13 for *S. superb* to 0.36 for *P. bournei*, leading to a negative allometric relationship between *M*_*L*_ and *M*_*R*_ across the entire data set (i.e., *M*_*L*_ ∝ *M*_*R*_^0.89 < 1.0^, 95% CI = 0.85–0.93) that differed significantly from 1.0 (*P* < 0.001).

**Figure 3:**
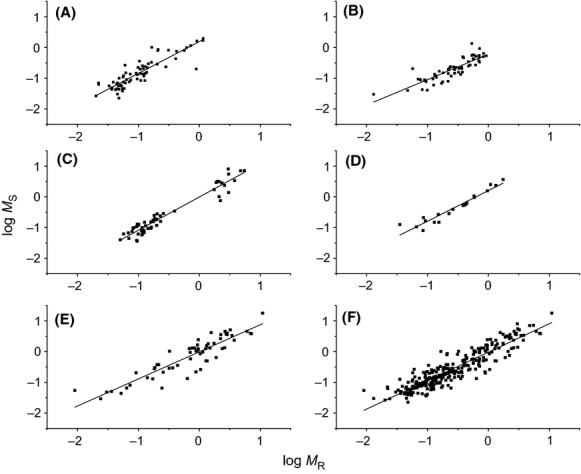
Log–log bivariate plots of stem versus root biomass (*M*_*S*_ vs. *M*_*R*_) within and across five evergreen tree species. (A) *Pinus massoniana*; (B) *Cunninghamia lanceolata*; (C) *Machilus pauhoi*; (D) *Phoebe bournei*; (E) *Schima superb*; (F) across five species.

Similarly, five species had the common slope for the relationship between stem and root biomass (i.e., *M*_*S*_ ∝ *M*_*R*_^1.06^, 95% CI = 1.01–1.18, *P* = 0.086) (Fig.3). Only two of five species had 95% CIs of the slopes that were slightly higher than unit (i.e., 1.01 and 1.04 for *P. massoniana* and *M. pauhoi*, respectively). Furthermore, across the entire data set, *M*_*R*_ scaled as 1.01-power of *M*_*S*_, which was indistinguishable from 1.0 (*P* = 0.683) (Table1; Fig.3).

### The scaling of *M*_*A*_ versus *M*_*R*_

**Figure 4:**
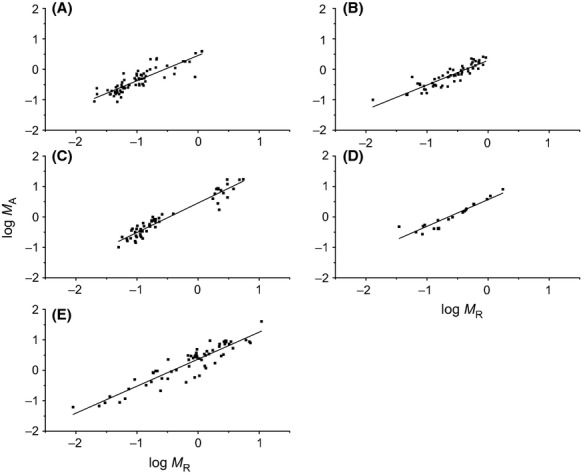
Log–log bivariate plots of above- versus below-ground (root) biomass (*M*_*A*_ vs. *M*_*R*_) within and across five evergreen tree species. (A) *Pinus massoniana*; (B) *Cunninghamia lanceolata*; (C) *Machilus pauhoi*; (D) *Phoebe bournei*; (E) *Schima superb*.

**Figure 5:**
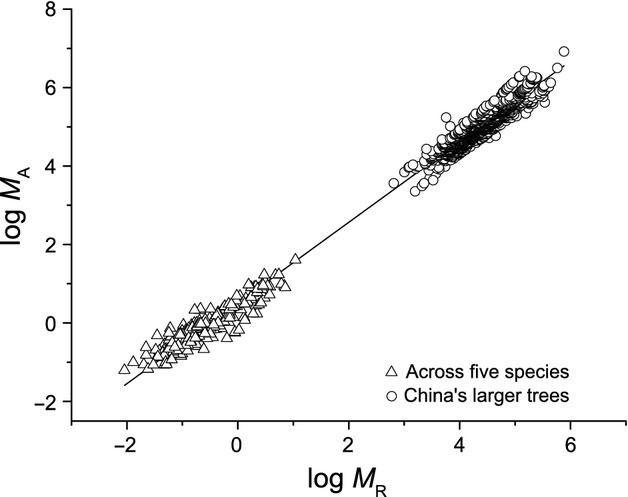
Log–log bivariate plots of above- versus below-ground (root) biomass (*M*_*A*_ vs. *M*_*R*_) across five evergreen tree saplings and the larger trees of China. The data of larger tree were taken from Luo (1996).

The above-ground biomass scaled isometrically with respect to below-ground biomass for five species, with a common slope of 0.97 (95% CI = 0.92–1.01, *P* = 0.65) (Table1; Figs.4, 5). The scaling constants ranged from 0.35 for *C. lanceolata* to 0.60 for *P. bournei*. Furthermore, across five species, the above-ground biomass scaled as 0.92 power with below-ground biomass, which was close to unity based on its 95% CIs (i.e., 0.88–0.96). Therefore, the variation in scaling constants of *M*_*A*_ versus *M*_*R*_ within five species did not change the isometric scaling exponent across the entire data sets.

**Figure 6:**
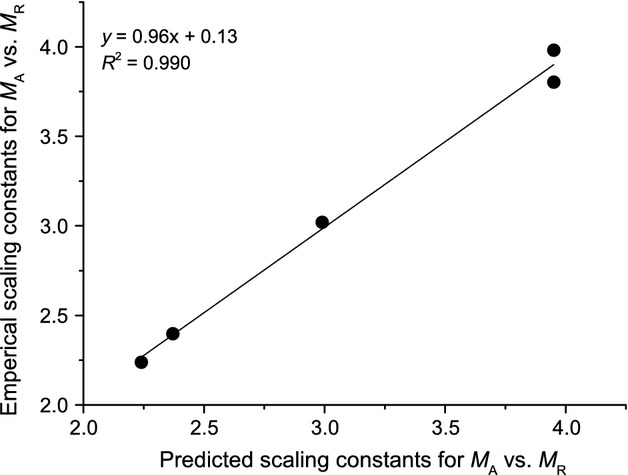
Bivariate plots of empirical and predicted scaling constants of above- versus below-ground (root) biomass (*M*_*A*_ vs. *M*_*R*_) for five evergreen species. The predicted scaling constants for *M*_*A*_ versus *M*_*R*_ were calculated from the scaling constants of leaf and stem versus root biomass through Eq. 2.

As expected from Eq. 2, the scaling constant for the scaling relationship of above- versus below-ground biomass should equal the sum of scaling constants of leaf and stem with respect to root (i.e., 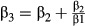, see Eq. 2). Such relationship was verified from the five woody species (Fig.6).

## Discussion

### Scaling relationships among leaf, stem and root biomass

Allometric theory predicted that *M*_*A*_ should scale nearly isometrically with *M*_*R*_ for small plants (e.g., seedlings) because of the isometric relationships existing among *M*_*L*_, *M*_*S,*_ and *M*_*R*_ (Eq. 1). As expected, our data indicated that isometric or near-isometric scaling relationships existed for *M*_*L*_ versus *M*_*R*_ and *M*_*S*_ versus *M*_*R*_ within five tree seedlings. However, our data did not support isometric scaling for *M*_*L*_ versus *M*_*S*_ in three of the five species and across the entire data sets.

Although RMA regression analyses of biomass allocation patterns indicated that scaling exponents of *M*_*L*_ versus *M*_*R*_ and *M*_*S*_ versus *M*_*R*_ were indistinguishable within and across the five species (*P* = 0.295 and 0.070, respectively), the *M*_*L*_ versus *M*_*R*_ scaling relationship excludes unique numerical values across five species, but not for within each species (Table1). Indeed, the five species of tree seedlings had a common slope of *M*_*L*_ versus *M*_*R*_ that is indistinguishable from 1.0 (Fig.2), whereas, across the entire data set, *M*_*L*_ scaled as 0.89-power with *M*_*R*_, which is significantly <1.0 (*P* < 0.001) (Table1; Fig.2). Therefore, we concluded that isometric scaling relationship between *M*_*L*_ versus *M*_*R*_ holds for intraspecific seedlings, but not for interspecific relationship. Further, we argued that the difference in the scaling constants for the relationship between *M*_*L*_ and *M*_*R*_ for five species (*P* < 0.01) (Table1), lead to the divergence in scaling exponents for intraspecies and interspecies.

The 95% CI for scaling exponents of *M*_*S*_ versus *M*_*R*_ for the five species include or near 1.0 (Table1; Fig.3). Furthermore, across the entire data set, *M*_*R*_ scaled as 0.99-power of *M*_*S*_, which is indistinguishable from 1.0 (*P* = 0.683). We conclude, therefore, that isometric scaling relationships for *M*_*S*_ versus *M*_*R*_ hold true within and across five species, irrespective the difference in scaling constants.

Niklas (2005) reported interspecific scaling relationships among leaf, stem, root, and above- to below-ground biomass for nonwoody plant and woody plant juveniles that lack secondary tissues (i.e., seedlings). Therefore, we also compared our data with such results. Specifically, the *M*_*L*_ versus *M*_*R*_ and *M*_*S*_ versus *M*_*R*_ regression slopes across small plants used to compare with our seedlings slopes were 0.94 (95% CIs = 0.92–0.98) and 0.98 (95% CIs = 0.95–1.02), respectively (Table1 in Niklas 2005). Although the data of Niklas (2005) collected most from nonwoody species, we have shown that the interspecific scaling exponents of *M*_*L*_ versus *M*_*R*_ and *M*_*S*_ versus *M*_*R*_ for woody seedlings were numerically consistent with that reported by Niklas (2005) based on the 95% CIs (Table1).

Interestingly, isometric or near-isometric scaling relationship existed for *M*_*L*_ ∝ *M*_*R*_, and *M*_*R*_ ∝ *M*_*S*_ within five species (Table1), leading us to speculate that such isometric scaling relationship might hold for *M*_*L*_ and *M*_*S*_ for each species. However, our data did not support this hypothesis (Table1; Fig.1). Prior studies have illustrated that the scaling exponents for leaf biomass and stem biomass range from 3/4 to 1.0, depending on the tree size (e.g., Enquist et al. 2007). The likely explanation for the systemic departure from isometry is that plants would allocate proportionately more to conducting and supporting tissues with increasing plant size (Niklas 2005; Mori et al. 2010). Our data indicated that the scaling exponents of *M*_*L*_ and *M*_*S*_ within and across five species all fell within such range. However, only two of five species (i.e., *C. lanceolata and S. superba*) exhibited isometric scaling relationship for *M*_*L*_ versus *M*_*S*_ as expected for seedlings. In addition, the scaling constants differed significantly among five species, resulting in a negative allometric relationship between *M*_*L*_ and *M*_*S*_ across the entire data (i.e. *M*_*L*_ ∝ *M*_*S*_^0.88^) (Table1; Fig.1). Indeed, the isometric hypothesis for *M*_*L*_ versus *M*_*S*_ is based on the suggestion of Reich et al. (2006) that scaling of metabolic rate in small plants is inherently isometric (Cheng et al. 2010; Mori et al. 2010; Peng et al. 2010) and that leaf is the only photosynthetic organ and one of the substitutions for plant metabolic rate (West et al. 1999; Enquist and Niklas 2002). Therefore, the departure from isometric scaling of three species may potentially be attributed to the fact that the leaf biomass is neither the only photosynthetic organ nor the good proxy for seedlings metabolism for three of five species. Firstly, as proposed by the functional equivalence hypothesis (FEH), the isometric biomass allocation for seedlings reflects the different parts (i.e., leaf, stem, and root) are functionally equivalent (Niklas 2006). Any change in one component should lead to the change in the other functional parts to maintain comparable functional levels of performance dictated by biophysically or physiologically invariant “rules”. According to FEH, it is reasonable to suspect that stem and leaf should be also functionally equivalent (i.e., *M*_*L*_ ∝ *M*_*S*_^1.0^) for seedlings to support rapid growth. Specifically, leaf has adapted to optimize photosynthesis, and stems elevate the leaves, serving as a conduit from the roots to the leaves. However, in addition to green leaves, stems of many plant species contain active chloroplasts, which efficiently perform photosynthetic CO_2_ assimilation (e.g., Aschan and Pfanz 2003; Teskey et al. 2008). Thus, according to FEH, a negative scaling exponent (i.e., scaling exponent <1.0) might be expected between *M*_L_ and *M*_S_ because the stem photosynthesis can contribute significantly to woody plant carbon balance. Secondly, the metabolic (e.g., respiration and photosynthesis) rates differ among different tissues and organs, as well as species (Ryan et al. 1994; Zha et al. 2004; Reich et al. 2008; Kutschera and Niklas 2012). Photosynthesis rate can vary according to resource allocation, and leaf age (Duursma et al. 2010); also photosynthetic tissues are not restricted to leaves (Deng et al. 2008; Koontz et al. 2009). Likewise, respiration rates vary nearly 40-fold among the different tissues of *Pinus strobes* (Vose and Ryan 2002). Therefore, the leaf might not be a good proxy of metabolism. Taken together, such deviations might account for the negative scaling relationships between leaf and stem for three of five species.

### Scaling relationship between above- and below-ground biomass

The relationships observed for *M*_*A*_ versus *M*_*R*_ were consistent with those predicted by the model for all five species. Unlike the invariant isometric scaling exponents, substantial variation in scaling constants was observed for five different species, indicating that absolute values of *M*_*A*_ vary substantially with respect to *M*_*R*_ across different species. That is *P. bournei* would had the highest stem to root ratio (SRR, scaling constant = 0.60) and *C. lanceolata* had the lowest SRR (scaling constant = 0.35). Our data also indicated that there is a nearly isometric relationship for *M*_*A*_ and *M*_*R*_ across the entire data, irrespective of the significant variations in allometric constant for different species (Table1; Fig.5). In addition, such interspecific isometric scaling was consistent with the results reported by Niklas (2005) that *M*_*A*_ scaled as 0.96 (95% CIs = 0.93–0.97) power with *M*_*R*_ for nonwoody plant based on 95% CIs. Moreover, the nearly isometric relationship between *M*_*A*_ and *M*_*R*_ observed in saplings is also agreement with pattern established in China's larger trees (Luo 1996) (Fig.5). Specifically, a scaling exponent of 1.02 (95% CIs = 1.02–1.03; *n* = 1524, *r*^2^ = 0.991) across saplings and larger trees is in agreement with isometric biomass allocation pattern expectations (e.g., Enquist and Niklas 2002), given that it is slightly larger than the predicted minimum value of 1.0. Therefore, we argue that nonwoody plant and seedlings of woody plants have the similar above- to below-ground biomass allocation scaling. Likewise, because the empirical scaling constants for *M*_*A*_ versus *M*_*R*_ were consistent with the predicted values within five species (Fig.6), our results provided support for FEH that above- and below-ground is be functionally equivalent (Niklas 2006).

It has long been acknowledged that above- and below-ground biomass allocation is influenced by the environment, plant size, competition and a variety of other factors (Brouwer 1962; Poorter et al. 2012a). Briefly, plants will allocate relatively more biomass to root if below-ground growth is limited (e.g., nutrients), whereas plants should allocate more biomass to shoot if above-ground growth is limited (e.g., light) (e.g., Davidson 1969; Hunt and Burnett 1973). However, such facts are accorded well with the allometric biomass partitioning studies because that the scaling constant represents the mean ratio above- to below-ground biomass (Gayon 2000). For example, previous studies indicate the plants growing under diverse environments had the nearly isometric scaling exponents between above- and below-ground biomass, but with different scaling constants (e.g., Cheng and Niklas 2007). Thus, the variations of the scaling constants in this studies reflect the intrinsic below- and above-ground biomass allocation properties among different species (Table1; Fig.4, 5). Further, another important factor regulating plant above- to below-ground biomass allocation is pot size effects (e.g., Bandara et al. 1998; Ray and Sinclair 1998; Hess and de Kroon 2007). Indeed, based on the meta-analysis, Poorter et al. (2012b) demonstrate that doubling of the pot size increases biomass production by 43%. Consistent with such findings, Hess and de Kroon (2007) assume that root size increases with pot size, regardless of nutrient concentration. However, based on the detailed study of *Cakile edentula*, Murphy et al. (2013) suggest that biomass allocation show complex pattern with pot size. That is, without increasing of nutrients, root biomass would do not increase with pot size. Therefore, whether the isometric allocation of above- and below-ground biomass holds true irrespectively the pot size effects remains to be seen. Therefore, future research toward understanding the scaling of plant biomass allocation requires special consideration of pot size effects.

## Conclusions

Isometric or nearly isometric scaling relationships were verified for leaf and stem with respect to root biomass, and thus above- to below-ground biomass for five woody species (i.e., *M*_*L*_ ∝ *M*_*R*_^*≈*1.0^, *M*_*S*_ ∝ *M*_*R*_^*≈*1.0^ and *M*_*A*_ ∝ *M*_*R*_^*≈*1.0^, respectively). However, statistically significant variation exists for scaling constants among five woody species for above scaling relationships. Although ANCOVA analyses indicated that intraspecific scaling exponents of *M*_*L*_ versus *M*_*R*_, *M*_*S*_ versus *M*_*R,*_ and *M*_*A*_ versus *M*_*R*_ were indistinguishable from the interspecific trend, the isometric scaling relationship does not hold for interspecific relationship for *M*_*L*_ versus *M*_*R*_, which is significantly <1.0 (i.e., *M*_*L*_ ∝ *M*_*R*_^0.89^). Nevertheless, variation in scaling constants leads to different scaling exponents for *M*_*L*_ versus *M*_*R*_, but nor for *M*_*S*_ versus *M*_*R*_ and *M*_*A*_ versus *M*_*R*_ within and across five evergreen woody species.

Furthermore, the negative scaling exponents were verified for three of five species and cross the entire data set for the relationship between *M*_*L*_ and *M*_*S*_ (*M*_*L*_ ∝ *M*_*S*_^<1.0^). We argue that stem photosynthesis violates the functional equivalence rule for plant biomass allocation, and that leaf might not be a good proxy of plant whole metabolism, resulting in the deviation from isometric scaling relationship. Thus, it requires additional data sets with which to compare our results. An investigation into how variation in the contribution of stem photosynthesis to seedling carbon balance affecting the scaling relationship between leaf and stem allocation for seedlings is particularly warranted.

## References

[b1] Aschan G, Pfanz H (2003). Non-foliar photosynthesis: a strategy of additional carbon acquisition. Flora.

[b2] Bandara MS, Tanino KK, Waterer DR (1998). Effect of pot size and timing of plant growth regulator treatments on growth and tuber yield in greenhouse-grown Norland and Russet Burbank potatoes. J. Plant Growth Regul.

[b3] Binkley D, Stape JL, Ryan MG (2004). Thinking about efficiency of resource use in forests. For. Ecol. Manage.

[b4] Brouwer R (1962). Distribution of dry matter in the plant. Neth. J. Agric. Sci.

[b5] Cheng DL, Niklas KJ (2007). Above- and below-ground biomass relationships across 1534 forested communities. Ann. Bot.

[b6] Cheng DL, Wang GX, Tang QL, Li T, Zhong QL (2009). Invariant allometric relationship between above- and below-ground biomass along a moisture gradient in North-West China. Pol. J. Ecol.

[b7] Cheng DL, Li T, Zhong QL, Wang GX (2010). Scaling relationship between tree respiration rates and biomass. Biol. Lett.

[b9] Davidson RL (1969). Effect of root/leaf temperature differentials on root/shoot ratios in some pasture grasses and clover. Ann. Bot.

[b10] Deng JM, Li T, Wang GX, Liu J, Yu ZL, Zhao CM (2008). Trade-offs between the metabolic rate and population density of plants. PLoS ONE.

[b11] Dodds P, Rothman DH, Weitz JS (2001). Reexamination of the ‘‘3/4-law’’ of metabolism. J. Theor. Biol.

[b12] Duursma RA, Mäkelä A, Reid DE, Jokela EJ, Porté AJ, Roberts SD (2010). Self-shading affects allometric scaling in trees. Funct. Ecol.

[b13] Enquist BJ, Niklas KJ (2002). Global allocation rules for patterns of biomass partitioning in seed plants. Science.

[b14] Enquist BJ, Allen AP, Brown JH, Gillooly JF, Kerkhoff AJ, Nikla KJ (2007). Biological scaling: does the exception prove the rule?. Nature.

[b15] Falster DS, Warton DI, Wright IJ (2003). http://www.bio.mq.edu.au/ecology/SMATR.

[b16] Gayon J (2000). History of the concept of allometry. Am. Zool.

[b17] Hess L, de Kroon H (2007). Effects of rooting volume and nutrient availability as an alternative explanation for root self/non-self discrimination. J. Ecol.

[b18] Hui DF, Jackson RB (2006). Geographical and interannual variability in biomass partitioning in grassland ecosystems: a synthesis of field data. New Phytol.

[b19] Hunt R, Burnett JA (1973). The effects of light intensity and external potassium level on root/shoot ratio and rates of potassium uptake in perennial ryegrass (*Lolium perenne* L.). Ann. Bot.

[b20] Koontz TL, Petroff A, West GB, Brown JH (2009). Scaling relations for a functionally two-dimensional plant: Chamaesyce Setiloba (Euphorbiaceae). Am. J. Bot.

[b21] Kozlowski J, Konarzewski M (2004). Is West Brown and Enquist's model of allometric scaling mathematically correct and biologically relevant?. Funct. Ecol.

[b22] Kutschera U, Niklas KJ (2012). Organ-specific rates of cellular respiration in developing sunflower seedlings and their bearing on metabolic scaling theory. Protoplasma.

[b23] Luo TX (1996). Patterns of biological production and its mathematical models for main forest types of China (in Chinese).

[b24] McCarthy MC, Enquist BJ (2007). Consistency between an allometric approach and optimal partitioning theory in global patterns of plant biomass allocation. Funct. Ecol.

[b25] Mori S, Yamaji K, Ishida A, Prokushkin SG, Masyagina OV, Hagihara A (2010). Mixed-power scaling of whole-plant respiration from seedlings to giant trees. Proc. Natl Acad. Sci. USA.

[b26] Murphy GP, File AL, Dudley SA (2013). Differentiating the effects of pot size and nutrient availability on plant biomass and allocation. Botany.

[b27] Niinemets U (1998). Growth of young trees of Acer platanoides and Quercus robur along a gap–understory continuum: interrelationships between allometry, biomass partitioning, nitrogen, and shade tolerance. Int. J. Plant Sci.

[b28] Niklas KJ (2004). Plant allometry: is there a grand unifying theory?. Biol. Rev.

[b29] Niklas KJ (2005). Modelling below- and above-ground biomass for nonwoody and woody plants. Ann. Bot.

[b30] Niklas KJ (2006). A phyletic perspective on the allometry of plant biomass and functional organ categories. New Phytol.

[b32] Peng Y, Niklas KJ, Reich PB, Sun S (2010). Ontogenetic shift in the scaling of dark respiration with whole-plant mass in seven shrub species. Funct. Ecol.

[b33] Poorter L (2001). Light-dependent changes in biomass allocation and their importance for growth of rainforest tree species. Funct. Ecol.

[b34] Poorter H, Niklas KJ, Reich PB, Oleksyn J, Poot P, Mommer L (2012a). Biomass allocation to leaves, stems and roots: meta-analyses of interspecific variation and environmental control. New Phytol.

[b35] Poorter H, Bühler J, van Dusschoten D, Climent J, Postma JA (2012b). Pot size matters: a meta-analysis of the effects of rooting volume on plant growth. Funct. Plant Biol.

[b36] Price CA, Enquist BJ, Savage VM (2007). A general model for allometric covariation in botanical form and function. Proc. Natl Acad. Sci. USA.

[b37] Ray JD, Sinclair TR (1998). The effect of pot size on growth and transpiration of maize and soybean during water deficit stress. J. Exp. Bot.

[b38] Reich PB, Tjoelker MG, Machado JL, Oleksyn J (2006). Universal scaling of respiratory metabolism, size and nitrogen in plants. Nature.

[b39] Reich PB, Tjoelker MG, Pregitzer KS, Wright IJ, Oleksyn J, Machado JL (2008). Scaling of respiration to nitrogen in leaves, stems, and roots of higher land plant. Ecol. Lett.

[b40] Ryan MG, Linder S, Vose JM, Hubbard RM (1994). Dark respiration of pines. Ecol. Bull.

[b41] Sack L, Marañón T, Grubb PJ (2002). Global allocation rules for patterns of biomass partitioning. Science.

[b42] Savage VM, Deeds EJ, Fontana W (2008). Sizing up allometric scaling theory. PLoS Comput. Biol.

[b43] Teskey RO, Saveyn A, Steppe K, McGuire MA (2008). Origin, fate and significance of CO_2_ in tree stems. New Phytol.

[b44] Vose JM, Ryan MG (2002). Seasonal respiration of foliage, fine roots and woody tissues in relation to growth, tissue N, and photosynthesis. Glob. Chang. Biol.

[b45] Wang X, Taub DR (2010). Interactive effects of elevated carbon dioxide and environmental stresses on root mass fraction in plants: a meta-analytical synthesis using pairwise techniques. Oecologia.

[b46] Warton DI, Weber NC (2002). Common slope tests for bivariate errors-in-variables. Biom. J.

[b47] Weiner J (2004). Allocation, plasticity and allometry in plants. Perspect. Plant Ecol.

[b48] West GB, Brown JH, Enquist BJ (1999). A general model for the structure and allometry of plant vascular systems. Nature.

[b49] West-Eberhard MJ (2003). Developmental plasticity and evolution.

[b50] Xue L, Lie G, Lu G, Shao Y (2013). Allometric scaling among tree components in *Pinus massoniana* stands with different sites. Ecol. Res.

[b51] Yang YH, Luo YQ (2011). Isometric biomass partitioning pattern in forest ecosystems: evidence from temporal observations during stand development. J. Ecol.

[b52] Yang YH, Fang JY, Ma WH, Guo DL, Mohammat A (2010). Large-scale pattern of biomass partitioning across China's grasslands. Glob. Ecol. Biogeogr.

[b53] Zha TS, Kellomaki Z, Wang KY, Ryyppo A, Niinisto S (2004). Seasonal and annual stem respiration of Scots pine trees under boreal conditions. Ann. Bot.

